# *BRAF*^*V600E*^ genetic testing should be recommended for Bethesda III or V thyroid nodules based on fine-needle aspiration

**DOI:** 10.1038/s41598-023-44464-1

**Published:** 2023-10-10

**Authors:** Yiran Lu, Xinghong Guo, Mengmeng Yang, Kewei Wang, Guanglei Cao, Yan Liu, Xinguo Hou, Li Chen, Kai Liang

**Affiliations:** 1 Department of Endocrine and Metabolic Diseases, Qilu Hospital of Shandong University, Jinan, China; 2 Institute of Endocrine and Metabolic Diseases of Shandong University, Jinan, China; 3 Key Laboratory of Endocrine and Metabolic Diseases, Shandong Province Medicine & Health, Jinan, China; 4 Jinan Clinical Research Center for Endocrine and Metabolic Diseases, Jinan, China

**Keywords:** Biotechnology, Cancer, Diseases, Endocrinology

## Abstract

The preoperative diagnosis of thyroid nodules now routinely includes *BRAF*^*V600E*^ genetic testing in most provincial and municipal hospitals in China. This study identified the most suitable patients of thyroid nodule for *BRAFV600E* genetic testing. We retrospectively collected data of patients from the Hospital Information System that had undergone fine needle aspiration biopsy (FNAB) from May 2019 to December 2021. Data of FNAB, *BRAF*^*V600E*^ genetic testing, and post-surgical pathological diagnosis were collected. A total of 12,392 patients were included in this study. Among them, 7,010 patients underwent solely FNAB, while 5,382 patients had both FNAB and *BRAF*^*V600E*^ genetic testing. In the FNAB group, 2,065 thyroid nodules were surgically removed, with a 93.12% malignancy rate. In the FNAB + BRAF group, 2,005 nodules were dissected, and the malignancy rate was 98.20%. However, it was evident that in the subgroups, the combination of FNAB and *BRAF*^*V600E*^ genetic testing only benefited Bethesda III (*p* < 0.001) and V (*p* = 0.001) nodules. Overall, the combination of FNAB with *BRAF*^*V600E*^ genetic testing significantly improved the malignancy rate of surgical thyroid nodes at our hospital when compared to FNAB alone. The subgroup analysis showed that *BRAF*^*V600E*^ genetic testing only benefited Bethesda III and V nodules. These findings provide a clinical reference for rationally selecting the most suitable population for *BRAF*^*V600E*^ genetic testing.

## Introduction

The incidence of thyroid cancer has gradually increased worldwide over the past few decades. Thyroid cancer became the 11th most prevalent type of cancer in 2020, with 580,000 new cases being recorded globally^1^. The mortality of thyroid cancer has rarely changed, even though the incidence and new cases are both increasing. As previously mentioned, thyroid cancer may be overdiagnosed^2,3^. How to balance the diagnostic and therapeutic approaches such that benign thyroid nodules and lower-risk patients undergo minimal essential identification and treatment is an urgent challenge that has to be solved for doctors.

Papillary thyroid cancer (PTC) is the most common type of thyroid cancer, accounting for more than 90% of thyroid cancer cases^4^. The most common gene mutation in PTC is the *BRAF*^*T1799A*^ mutation, which ultimately results in the *BRAF*^*V600E*^ mutation^5^. B-type Raf kinase (BRAF) is a serine/threonine protein kinase, whose mutation helps to activate the mitogen-activated protein kinase (MAPK) signal transduction pathway^6^. *BRAF*^*V600E*^ mutation is observed in about 43–88% PTC, 20–40% poorly differentiated thyroid cancer^7–11^, and 30–40% anaplastic thyroid cancer^12^. Clinically, the detection of preoperative or postoperative *BRAF* mutation exhibits a great impact on the diagnosis, prognostic stratification, and treatment of PTC. Nowadays, fine needle aspiration (FNA) can be done to obtain samples from the thyroid nodules for cytopathology and *BRAF* mutation detection. Many studies indicated that genetic testing of thyroid FNA samples greatly enhanced diagnostic accuracy and prognostication^13,14^. The final medical decision would be made by doctors and surgeons based on the features of medical imaging as well as the FNA cytopathological and genetic results.

The most suitable population of thyroid nodule for *BRAF* mutation detection must be identified to avoid overdiagnosis of thyroid cancer. The 2015 American thyroid association management guidelines for adult patients with thyroid nodules and differentiated thyroid cancer stated that molecular testing may be used to supplement malignancy risk assessment for nodules of atypia of undetermined significance (AUS)/ follicular lesion of undetermined significance (FLUS) (Bethesda III), follicular neoplasm (FN)/ suspicious for a follicular neoplasm (SFN) (Bethesda IV) and suspicious for malignancy (SFM) (Bethesda V)^15,16^. American Association of Clinical Endocrinologists/American College of Endocrinology/Associazione Medici Endocrinologi guidelines suggested that molecular testing could be performed for nodules that lacked established benign or malignant cytologic characteristics. Notably, *BRAF* mutation could guide to determine the scope of surgery^17^. According to European thyroid association guidelines, *BRAF* and *RET/PTC* mutations, as well as possibly *PAX8/PPARG* and *RAS* mutations, should be considered for cytologically indeterminate nodules, if available^18^. The Bethesda III and V nodules may benefit more from *BRAF* mutation detection because thyroid follicular neoplasm (Bethesda IV) cases with *BRAF* mutation are substantially less common^19^.

Since May 2019, we established *BRAF*^*V600E*^ genetic testing based on fine-needle aspiration in Qilu Hospital, Shandong University. Here, we conducted a retrospective cohort study including 12,392 individuals who received FNA cytologic Bethesda grading with or without *BRAF*^*V600E*^ genetic testing to determine the most suitable population for the test. We found that Bethesda III and V nodules were the only ones who benefited from *BRAF*^*V600E*^ genetic testing as a supplement to FNA. Our findings provided a large database that may be used for rationally selecting the most suitable population for *BRAF* genetic testing. Additionally, our data provided a basis for avoiding overdiagnosis of thyroid nodule cases.

## Methods

### Study population

This study was conducted in accordance with the Declaration of Helsinki and approved by the ethics committee of Qilu Hospital of Shandong University (ethical approval number KYLL-2018(KS)-226). The ethics committee of Qilu Hospital of Shandong University has approved that since only existing anonymized data were used in this study, it is not necessary to obtain the informed consent of each individual. We retrospectively collected data from all patients who underwent fine needle aspiration biopsy (FNAB), with or without *BRAF*^*V600E*^ genetic testing, and post-surgical pathological diagnosis from the Hospital Information System of Qilu Hospital of Shandong University from May 2019 to December 2021. A total of 12,392 patients were included in this study. The *BRAF*^*V600E*^ genetic testing and treatment plan were determined according to doctors' experience and patients' wishes. Among them, 7,010 patients underwent solely FNAB (FNAB group) and 5,382 patients underwent both FNAB and *BRAF*^*V600E*^ genetic testing (FNAB + BRAF group). A total of 2,065 nodules in the FNAB group and 2,005 nodules in the FNAB + BRAF group were removed by surgery.

### Cytology, *BRAF*^*V600E*^ genetic testing and post-surgical pathological diagnosis

The procedure of FNAB was described in detail previously^20^. Cytological classification was based on The Bethesda System for Reporting Thyroid Cytopathology^16^. Real-time polymerase chain reaction (RT-PCR) was used for *BRAF*^*V600E*^ genetic testing. The puncture tissue was stored in a 1.5 mL eppedorf tube containing 200µL lysate buffer and sent to the Department of Pathology for analysis. The *BRAF p.V600E* Mutations Detection Kit (Amoy Diagnostics Co., Ltd., Xiamen, China) was used for real-time PCR analysis. Primers and probes sequences are listed in Table 1. Post-surgical pathological diagnosis was based on the WHO Classification of Tumours of Endocrine Organs^21^.

**Table 1:** Primer sequences and probe sequences.

	Sequences
Primer	
B-raf-F-tag	tctgtagcAGCCCTCAGTAGCGAAGCAGTGATTTTGGTCTAGCTACAGA
B-raf-R-tag	AGCCCTCAGTAGCGAAGCAACTCAGCAGCATCTCAGG
T-primer	AGCCCTCAGTAGCGAAGCA
Exon-2-S10-tag	AGCCCTCAGTAGCGAAGCAGCACGAGTAACAAGCTCACG
Exon-2-R10-tag	AGCCCTCAGTAGCGAAGCAGATCATAATTCCTCTGCACATAGGTAA
Probe	
B-raf-P–C	FAM-5ˊ-TTCAAACCATCAGTTTGAACAGTTGTCTGGATCAACTG-3ˊ- Tamra
Ex-2-P–C	HEX-5ˊ-CTCTGGACAGCCTCCAGAGGATGTTCAATAACTGAACATC-3ˊ-BHQ2

### Statistical analysis

Frequencies and percentages reported for categorical variables. Differences in frequencies were analyzed via the chi-square test. Two-tailed *p* < 0.05 was considered statistically significant. Sensitivity, specificity, positive predictive value (PPV), negative predictive value (NPV), and accuracy were calculated for each detection method and combined methods, considering histology as the gold standard. All data were analyzed using SPSS 22.0 (SPSS Inc., Chicago, IL, USA) software.

### Ethics approval

This study was approved by the ethics committee of Qilu Hospital of Shandong University (ethical approval number KYLL-2018(KS)-226).

## Results

### Characteristics of study participants

A total of 12,392 patients were included in this study. Among them, 7010 patients underwent only FNAB, while 5382 patients underwent both FNAB and *BRAF*^*V600E*^ genetic testing. A total of 2065 thyroid nodules in the FNAB group underwent surgical removal, with a 93.12% malignancy rate. In the FNAB + BRAF group, 5469 nodules were aspirated for *BRAF*^*V600E*^ genetic testing, and 3113 (56.92%) of them tested positive for the mutation, and a total of 2005 nodules were dissected with a malignancy rate of 98.20%. We analyzed the positive rates of *BRAF*^*V600E*^ genetic testing in different cytology Bethesda groups (Table 2, demographic characteristics in Table S1).

**Table 2:** Positive rates of *BRAF*^*V600E*^ genetic testing in different cytology Bethesda groups.

Bethesda groups	Number of nodules	Number of nodules with *BRAF*^*V600E*^ positive	Positive rates
I	259	19	7.34%
II	1332	17	1.28%
III	506	139	27.47%
IV	73	1	1.37%
V	287	204	71.08%
VI	3012	2733	90.74%
Total	5469	3113	56.92%

### Cytopathological examination and ***BRAF***^***V600E***^ analysis

**Figure 1:**
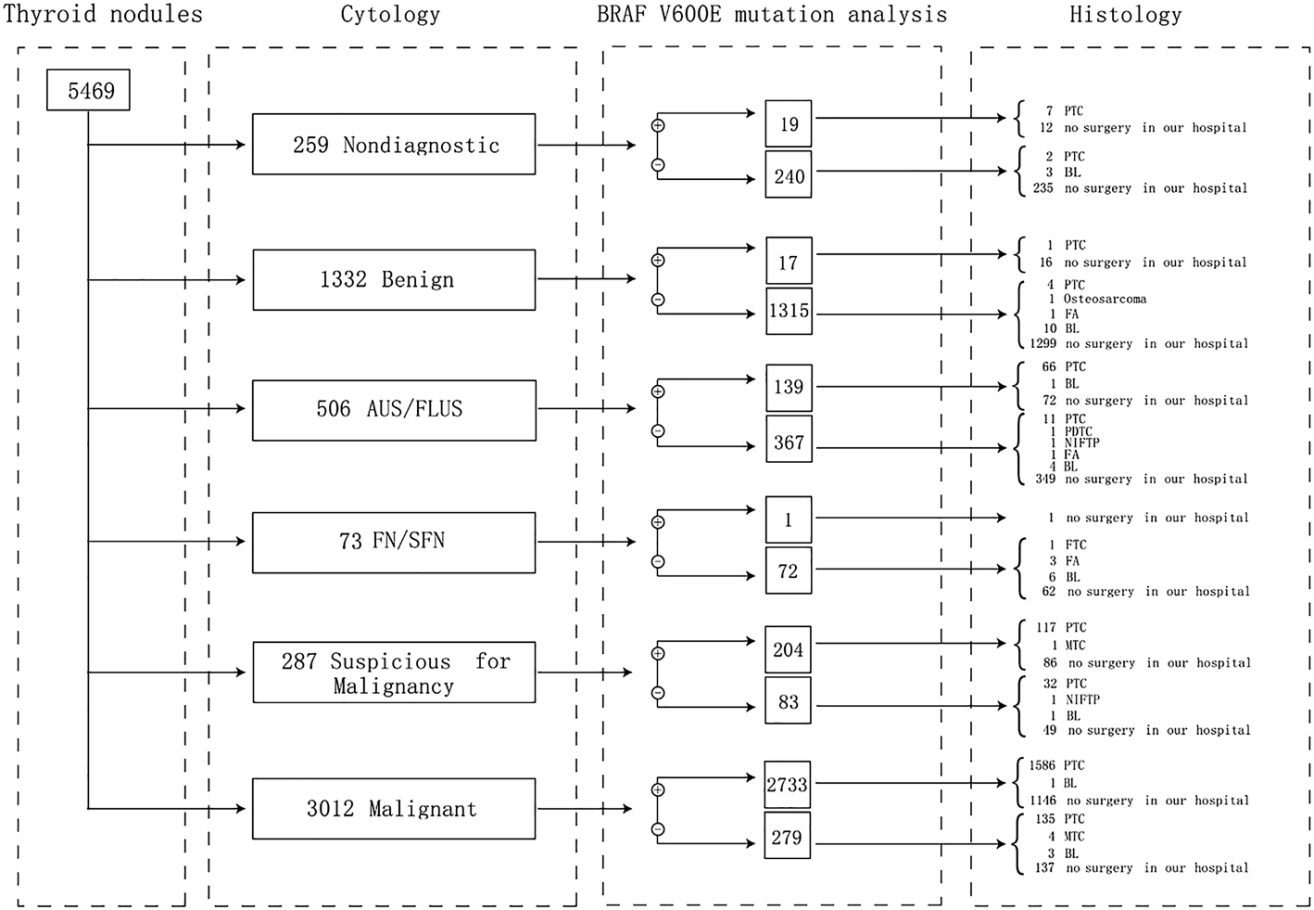
Cytology, *BRAF*^*V600E*^ mutation and histology of thyroid nodules with *BRAF*^*V600E*^ genetic testing.

The cytopathological results of the 5469 nodules that underwent *BRAF*^*V600E*^ genetic testing are depicted in Fig. 1. The *BRAF*^*V600E*^ mutation was identified in 3113/5469 nodules, and 2937 of these nodules were identified as malignant or SFM by cytology. In the remaining 176 *BRAF*^*V600E*^ mutant nodules, cytology was consistent with 19 nondiagnostic, 17 benign, 139 AUS/FLUS, and 1 FN/SFN.

The 7 *BRAF*^*V600E*^ mutant patients out of the 19 patients who had nondiagnostic cytology underwent surgery in our hospital, and all of the post-surgical pathologies were determined as PTC. One *BRAF*^*V600E*^ mutant patient with benign cytology underwent surgery and the pathology confirmed PTC. Out of the 139 *BRAF*^*V600E*^ mutant patients with AUS/FLUS cytology, 67 underwent surgery, 66 were determined to be PTC, and the remaining patients had benign lesions. The only one *BRAF*^*V600E*^ mutant patient of the 73 with FN/SFN cytology did not underwent thyroid surgery in our hospital.

### Diagnostic value of cytology and ***BRAF***^***V600E***^ mutation analysis

We collected the post-surgical data to determine the diagnostic significance of cytology and *BRAF*^*V600E*^ genetic testing. The overall malignancy rates of Bethesda I to VI were 75.00%, 35.29%, 91.76%, 10.00%, 98.68% and 99.77%. The malignancy rates of Bethesda I to VI in those *BRAF*^*V600E*^ mutant patients were 100.00%, 100.00%, 98.51%, None, 100% and 99.94%. The malignancy rates of Bethesda I to VI in patients without *BRAF*^*V600E*^ mutation were 40.00%, 31.25%, 66.67%, 10.00%, 94.12% and 97.89% (Table 3, demographic characteristics in Table S2). As shown in Table 4, patients in the FNAB + BRAF group who underwent surgical resection, either Cytology or *BRAF*^*V600E*^ positive showed higher sensitivity (98.98%), NPV (59.18%) and accuracy (98.65%). Nevertheless, both cytology and *BRAF*^*V600E*^ positive exhibited higher specificity (97.22%) and high PPV (99.94%).

**Table 3:** Post-surgical malignancy rate of thyroid nodules with or without *BRAF*^*V600E*^ mutation in FNAB + BRAF group.

Bethesda groups	Total			*BRAF*^*V600E*^ positive			*BRAF*^*V600E*^ negative		
	Number of surgical nodules	Number of malignant nodules	Malignancy rate	Number of surgical nodules	Number of malignant nodules	Malignancy rate	Number of surgical nodules	Number of malignant nodules	Malignancy rate
I	12	9	75.00%	7	7	100.00%	5	2	40.00%
II	17	6	35.29%	1	1	100.00%	16	5	31.25%
III	85	78	91.76%	67	66	98.51%	18	12	66.67%
IV	10	1	10.00%	0	0	0	10	1	10.00%
V	152	150	98.68%	118	118	100.00%	34	32	94.12%
VI	1729	1725	99.77%	1587	1586	99.94%	142	139	97.89%
Total	2005	1969	98.20%	1780	1778	99.89%	225	191	84.89%

**Table 4:** Diagnostic performance of cytology and *BRAFV600E* genetic testing in FNAB + BRAF group.

	Cytology positive	*BRAF*^*V600E*^ positive	Either Cytology or *BRAF*^*V600E*^ positive	Both Cytology and *BRAF*^*V600E*^ positive
Sensitivity	95.23%	90.30%	98.98%	86.54%
Specificity	83.33%	94.44%	80.56%	97.22%
Positive predictive value	99.68%	99.89%	99.64%	99.94%
Negative predictive value	24.19%	15.11%	59.18%	11.67%
Accuracy	95.01%	90.37%	98.65%	86.73%

### Clinical benefits of the combination of cytology and ***BRAF***^***V600E***^ genetic testing

It is essential to better understand which patients can benefit from the combination of cytology and *BRAF*^*V600E*^ genetic testing to reduce overdiagnosis of thyroid nodules. For all nodules, the combination enhanced the malignancy rate of the nodules that were destined to surgery(*p* < 0.001). However, in the subgroup analysis, the combination only benefited Bethesda III (*p* < 0.001) and V (*p* = 0.001) (Table 5, demographic characteristics in Table S3).

**Table 5:** Post-surgical malignancy rate of FNAB and FNAB with *BRAFV600E* genetic testing in different cytology Bethesda groups.

Bethesda groups	FNAB alone			FNAB with *BRAF*^*V600E*^ genetic testing			*p*
	Number of surgical nodules	Number of malignant nodules	Malignancy rate	Number of surgical nodules	Number of malignant nodules	Malignancy rate	
I	16	8	50.00%	12	9	75.00%	0.253
II	68	19	27.94%	17	6	35.29%	0.563
III	63	32	50.79%	85	78	91.76%	< 0.001
IV	33	9	27.27%	10	1	10.00%	0.407
V	194	175	90.21%	152	150	98.68%	0.001
VI	1691	1680	99.35%	1729	1725	99.77%	0.073
Total	2065	1923	93.12%	2005	1969	98.20%	< 0.001

## Discussion

The prevalence of thyroid cancer has increased significantly as people's health awareness and screening techniques have advanced^22^, yet mortality have remained consistently low^23,24^, indicating that there may be overdiagnosis and overtreatment of thyroid cancer^25^. Therefore, timely evidence of the epidemiological situation is needed to determine the magnitude of the problem and suggest workable solutions. The use of FNAB has significantly improved the accuracy of clinical diagnosis of thyroid nodules, and it is highly recommended as the gold standard for preoperative differentiation of benign and malignant thyroid nodules throughout the world^15^. Although most thyroid nodules can be accurately identified as benign or malignant by FNAB, 10%-30% of thyroid nodules are unable to be identified by FNAB and are classified as AUS/FLUS, FN/SFN or SFM^16,26,27^. Additionally, studies have shown that about 70–80% of these indeterminate nodules are eventually proven to be benign, allowing surgery to be avoided^28^. Uncertainty in cytology can lead to conflicting clinical decisions, which may result in unnecessary surgery or delay in the treatment of the disease.

With the rapid development of molecular biology, genetic tests have played an important role in the identification of benign and malignant thyroid nodules, and have gradually become the focus of researchers and clinicians^29,30^. The conventional basis for accurate diagnosis for FNAB will depend on certain experienced cytologists. While liquid based biopsy further improved the accuracy in comparison with crush cytology. Therefore, it will be expected that adding on genetic testing might further more increase the success rate of correct diagnosis pre-operatively. BRAF, a member of the MAPK pathway, is located on chromosome 7q34 and is a cytoplasmic serine-threonine protein kinase, which can cause phosphorylation and activation of the MAPK pathway, which is involved in cell metabolism, proliferation, differentiation, apoptosis, migration, and other processes^31^. *BRAF*^*V600E*^ mutation is common in PTC, poorly differentiated thyroid cancer, and anaplastic thyroid cancer, and rare in follicular thyroid cancer, medullary thyroid cancer, benign thyroid adenoma, or hyperplasia^32^. According to research, *BRAF* mutations are found in roughly 43–88% of PTC^7–11^, making it the most frequent mutation in PTC. It is directly associated with aggressive tumor behavior and poor clinical outcome of PTC^33^. This phenomenon appears to be more pronounced in East Asian populations^34^. Due to the high frequency of *BRAF*^*V600E*^ mutation in PTC, *BRAF*^*V600E*^ mutation detection for diagnostic purposes has shown high sensitivity and specificity for determining the presence of tumor cells^35^. As a result, many provincial and municipal medical institutions in China have included *BRAF*^*V600E*^ genetic testing as a routine item in the preoperative diagnosis of thyroid nodules in recent years, and overdiagnosis may be present^3^. However, it does not imply that all samples should be subjected to genetic testing. For specimens where a conclusive diagnosis can be made by cytology, genetic tests are not necessary. The latest version of The Bethesda System for Reporting Thyroid Cytology (TBSRTC) specifies that genetic tests should be used in FNA samples of thyroid nodules with an indeterminate diagnosis^15,16^. An important scientific question that needs to be seriously studied at the moment is how to prevent blindly following the trend in this surge and accomplish the sensible development and usage of genetic tests. Therefore, we conducted this study in an attempt to find a more suitable condition for the *BRAF*^*V600E*^ genetic test to provide a basis for standardizing the diagnosis and treatment of thyroid nodules.

In this retrospective study, we determined the *BRAF*^*V600E*^ mutation rate in thyroid nodules with different Bethesda grades of FNA cytology. The highest *BRAF*^*V600E*^ mutation rate was found in Bethesda VI nodules, 90.74%; while the lowest *BRAF*^*V600E*^ mutation rate was found in Bethesda II nodules, 1.28%. It was similar to the previous study by Chen et al. and correlated with the degree of benignity and malignancy of the nodules^35^. Additionally, very low (1.37%) of Bethesda IV nodules had *BRAF*^*V600E*^ mutations, which was consistent with the characteristics of gene mutation in thyroid follicular tumors^10^. We analyzed 5469 thyroid nodules from patients that underwent both FNAB and *BRAF*^*V600E*^ genetic testing, out of which 2005 underwent thyroid surgery at our institution, with a postoperative pathological malignancy rate of 98.20%, which was higher than that of patients who underwent FNAB alone and thyroid surgery during the same period (98.20% vs. 93.12%, *p* < 0.001), indicating that *BRAF*^*V600E*^ genetic testing can assist the preoperative diagnosis of thyroid nodules. Especially for Bethesda III nodules, whether or not to perform thyroid lobectomy has become a vexing one for clinicians. Aggressive thyroid lobectomy may cause unnecessary trauma and increase the financial burden of the patient. This study showed that the postoperative pathological malignancy rate of Bethesda III nodules with positive *BRAF*^*V600E*^ mutation reached 98.51%. Therefore, for Bethesda III nodules, combined with *BRAF*^*V600E*^ genetic testing can provide a better basis for whether to perform a thyroid lobectomy.

We evaluated the diagnostic efficacy of cytology, *BRAF*^*V600E*^ genetic testing, and their combination for the identification of benign and malignant thyroid nodules. *BRAF*^*V600E*^ genetic testing alone performed better in terms of specificity and PPV for the identification of benign and malignant thyroid nodules, but it had lower sensitivity, NPV, and accuracy than cytology. This meant that in samples with an indeterminate diagnosis based on FNAB cytology, a positive *BRAF*^*V600E*^ mutation suggested that the nodule was extremely likely to be PTC, but it was not sensitive enough in those with negative mutations to exclude the diagnosis of malignancy. When either cytology or *BRAF*^*V600E*^ was positive, there was high sensitivity and NPV, which is a good diagnostic test for exclusion, and a negative result was accurate enough to ensure that the thyroid nodule was not malignant. When both cytology and *BRAF*^*V600E*^ were positive, there was high specificity and PPV, indicating that the test is a solid confirmatory diagnostic test, and a positive result confirmed nodule malignancy.

It is widely accepted that enhancing the sensitivity and specificity of diagnostic methods for thyroid nodules is an effective way to minimize unnecessary surgery and overtreatment^36^. Overall, the combination of FNAB with the *BRAF*^*V600E*^ genetic test at our hospital significantly improved the malignancy rate of surgical nodes compared to the FNAB alone. Because of the high sensitivity of cytology alone and the high specificity of BRAFV600E gene testing alone, there was a high sensitivity (98.98%) to identify the benign and malignant tumors by either cytological or BRAFV600E positivity, and high specificity (97.22%) by both cytological and BRAFV600E positivity. Clinically, we prefer high-sensitivity detection methods allow for early detection and removal of malignant nodules, resulting in a favorable prognosis for the patients.

However, a subgroup analysis of thyroid nodules of different Bethesda classifications revealed that the *BRAF*^*V600E*^ genetic test was more appropriate for Bethesda III or V thyroid nodules and ineffective in Bethesda I, II, IV, and VI thyroid nodules. Therefore, expanding the detection of biomarkers of thyroid nodules blindly may lead to overtreatment and waste of medical resources. As a result, we recommend that patients with Bethesda III or V thyroid nodules perform FNA again to test *BRAF*^*v600E*^ mutations, rather than conducting cytology combined with *BRAF*^*v600E*^ gene testing for all patients. A recent study showed that the *BRAF*^*V600E*^ mutation can be detected in FNA residual samples using a locked nucleic acid probe–based droplet digital polymerase chain reaction without using fresh puncture samples^27^. Therefore, *BRAF*^*V600E*^ mutation may be detected in FNA residual samples for thyroid nodules with cytologically determined Bethesda III or V in the future.

In addition to the diagnosis of thyroid cancer, the *BRAF*^*V600E*^ genetic test can aid in the development of treatment strategies for thyroid cancer patients^37^. If the *BRAF*^*V600E*^ mutation is detected before treatment, it suggests a more aggressive tumor that requires a more extensive surgical option^38–40^. However, given the low-risk characteristics of most PTC in the clinic, genetic testing is expected to influence diagnosis and treatment decisions, but it cannot provide predictive power beyond Tumor Node Metastasis staging. The development of an American Thyroid Association risk stratification system remains the best way to predict the future recurrence of disease after initial treatment^15^. The future of genetic testing in thyroid pathology remains extremely promising and it has the potential to affect not just thyroid nodule diagnosis and surgery, but also the role of radioiodine therapy in the treatment and other possible systemic therapies.

Our study had the following limitations. Only the malignant rate of surgical pathology was analyzed, without subdivision of surgical methods, thyroid cancer classification, size, and number of lesions, cervical lymph nodes, or distant metastases. The enrolled samples had a high rate of postoperative histopathological malignancy. However, we enrolled all patients with FNAB + BRAF from May 2019 to December 2021, which to some extent avoided sample selection bias and also reflected the high accuracy of our hospital (as the largest general hospital in Shandong Province) in the diagnosis of thyroid nodules. We only counted the post-surgical pathological diagnosis of patients who underwent surgery at our hospital. Since our hospital is a regional medical center, patients are willing to come to our hospital to clarify the diagnosis and determine the treatment plan. But due to the limited medical resources of our hospital, some patients will go to other hospitals for surgery after clarifying the diagnosis and treatment plan, and the post-surgical pathological diagnosis of these patients were not included in this study.

We performed a retrospective cohort study including 12,392 patients who had FNA cytologic Bethesda grading with or without *BRAF*^*V600E*^ genetic testing. This is a real-world study, which can reflect the application value of *BRAF*^*V600E*^ genetic testing for the identification of benign and malignant thyroid nodules before surgery. We found that the addition of *BRAF*^*V600E*^ genetic testing benefited only Bethesda III and V nodules. Our findings provided a large database that may be used for rationally selecting the most suitable population for *BRAF*^*V600E*^ genetic testing. Additionally, our data provided a basis for avoiding overdiagnosis in thyroid nodule patients.

### Supplementary Information

Supplementary Information.

## Data Availability

De-identified individual data might be available following publication by reasonable request to the corresponding author accompanied by research proposal.
