# Supplementary material for: BRAFV600E genetic testing should be recommended for Bethesda III or V thyroid nodules based on fine-needle aspiration
Source: Sci Rep. 2023 Oct 10;13:17129. doi: 10.1038/s41598-023-44464-1 (PMC10564976; doi:10.1038/s41598-023-44464-1)
Supplement: Supplementary file 1 — Supplementary Information. [file 41598_2023_44464_MOESM1_ESM.docx]

**Table S1** The demographic data of different cytology Bethesda groups in FNAB+BRAF group

| Bethesda groups | Number of nodules | Age (years) | Female (%) |
| --- | --- | --- | --- |
| I | 259 | 49.63±11.35 | 74.13% |
| II | 1332 | 50.79±11.85 | 81.46% |
| III | 506 | 47.45±12.08 | 77.87% |
| IV | 73 | 45.63±13.79 | 73.97% |
| V | 287 | 45.17±11.34 | 78.05% |
| VI | 3012 | 43.86±11.57 | 75.37% |
| Total | 5469 | 46.25±12.07 | 77.14% |

**Table S2** The demographic data of post-surgical patients with or without *BRAF^V600E^* mutation in FNAB+BRAF group

| Bethesda groups | Total | | |  | *BRAF^V600E^* positive | | |  | *BRAF^V600E^* negative | | |
| --- | --- | --- | --- | --- | --- | --- | --- | --- | --- | --- | --- |
|  | Age (years) | Female (%) | Maximum diameter of nodule (cm) |  | Age (years) | Female (%) | Maximum diameter of nodule (cm) |  | Age (years) | Female (%) | Maximum diameter of nodule (cm) |
| I | 47.25±13.42 | 58.33% | 0.6(0.6-1.23) |  | 42.14±13.83 | 57.14% | 0.6(0.6-0.6) |  | 54.40±9.91 | 60% | 1.3(0.7-2.15) |
| II | 53±14.12 | 82.35% | 1.5(0.7-4.35) |  | 63 | - | 1.5 |  | 52.38±14.33 | 87.5% | 1.95(0.65-4.43) |
| III | 47.08±10.18 | 74.12% | 0.6(0.4-.95) |  | 46.57±10.27 | 74.63% | 0.5(0.4-0.8) |  | 49.00±9.89 | 72.22% | 0.95(0.5-1.83) |
| IV | 37.6±15.35 | 60% | 2.7(2-4) |  | - | - | - |  | 37.60±15.35 | 60% | 2.7(2-4) |
| V | 45.65±11.58 | 76.97% | 0.5(0.4-0.8) |  | 46.37±11.00 | 80.51% | 0.5(0.4-0.7) |  | 43.15±13.28 | 64.71% | 0.75(0.5-1.5) |
| VI | 43.82±11.28 | 74.49% | 0.7(0.5-1) |  | 43.94±11.24 | 74.92% | 0.7(0.5-1) |  | 42.51±11.72 | 69.72% | 0.8(0.5-1.3) |
| Total | 44.17±11.37 | 74.56% | 0.7(0.5-1) |  | 44.20±11.22 | 75.17% | 0.7(0.5-1) |  | 42.88±12.554 | 69.78% | 0.8(0.55-1.5) |

**Table S3** The demographic data of post-surgical patients in FNAB or FNAB+BRAF groups in different cytology Bethesda groups

| Bethesda groups | FNAB alone | | |  | FNAB with *BRAF^V600E^* genetic testing | | |  |
| --- | --- | --- | --- | --- | --- | --- | --- | --- |
|  | Age (years) | Female (%) | Maximum diameter of nodule (cm) |  | Age (years) | Female (%) | Maximum diameter of nodule (cm) |  |
| I | 47.63±9.99 | 87.5% | 0.7(0.5-1.5) |  | 47.25±13.12 | 58.33% | 0.6(0.6-1.23) |  |
| II | 50.59±12.18 | 77.94% | 1.05(0.63-2.78) |  | 53±14.12 | 82.35% | 1.5(0.7-4.3) |  |
| III | 47.33±11.81 | 71.43% | 0.7(0.5-1.6) |  | 47.08±10.18 | 74.12% | 0.6(0.4-0.95) |  |
| IV | 45.85±14.02 | 69.7% | 0.9(0.6-1.85) |  | 37.6±15.35 | 60% | 2.7(2-4) |  |
| V | 46.75±12.03 | 78.53% | 0.7(0.5-1.0) |  | 45.65±11.58 | 76.97% | 0.5(0.4-0.8) |  |
| VI | 44.61±11.82 | 74.14% | 0.8(0.6-1.2) |  | 43.82±11.28 | 74.49% | 0.7(0.5-1) |  |
| Total | 45.13±11.93 | 74.62% | 0.8(0.6-1.2) |  | 44.17±11.37 | 74.56% | 0.7(0.5-1) |  |
